# Behavior of Centromeres during Restitution of the First Meiotic Division in a Wheat–Rye Hybrid

**DOI:** 10.3390/plants11030337

**Published:** 2022-01-27

**Authors:** Adam J. Lukaszewski

**Affiliations:** Department of Botany and Plant Sciences, University of California, Riverside, CA 92521, USA; adam.lukszewski@ucr.edu

**Keywords:** meiosis, restitution of the first division, centromere, karyokinetic spindle

## Abstract

In first division restitution (FDR)-type meiosis, univalents congregate on the metaphase I plate and separate sister chromatids in an orderly fashion, producing dyads with somatic chromosome numbers. The second meiotic division is abandoned. The separation of sister chromatids requires separation of otherwise fused sister centromeres and a bipolar attachment to the karyokinetic spindle. This study analyzed packaging of sister centromeres in pollen mother cells (PMCs) in a wheat–rye F_1_ hybrid with a mixture of standard reductional meiosis and FDR. No indication of sister centromere separation before MI was observed; such separation was clearly only visible in univalents placed on the metaphase plate itself, and only in PMCs undergoing FDR. Even in the FDR, PMCs univalents off the plate retained fused centromeres. Both the orientation and configuration of univalents suggest that some mechanism other than standard interactions with the karyokinetic spindle may be responsible for placing univalents on the plate, at which point sister centromeres are separated and normal amphitelic interaction with the spindle is established. At this point it is not clear at all what univalent delivery mechanism may be at play in the FDR.

## 1. Introduction

In haploids or wide hybrids of plants, standard meiosis can sometimes be abbreviated to a single division, generating two products instead of four, but with somatic chromosome numbers. This phenomenon has been observed and described numerous times in haploids and wide hybrids of wheat, *Triticum aestivum* L. [1,2,3,4,5,6]. It effectively doubles the chromosome number of progeny; a mechanism probably responsible in large part for the creation of allopolyploids [7]. Several different terms have been proposed for such altered meiosis: “unreductional meiotic cell division”, “single division meiosis”, “haploid meiotic restitution” or “indeterminate meiotic restitution” [5,8,9,10,11]. In some situations there appears to be no division at all with the resulting monad carrying twice the somatic chromosome number of the generating plant [1]. For the sake of consistency with general terminology [8], the term “first division restitution”, FDR, will be used here. In FDR, univalents line up in an orderly fashion on the metaphase plate of what should be the first meiotic division and separate sister chromatids in anaphase I; second division is abandoned. The final product of FDR is a dyad where each nucleus, in most cases, contains a somatic number of chromosomes. Occasional chiasmate pairing, or precocious migration of some univalents to the poles, alter the chromosome constitutions of the gametes and of resulting progeny [12,13,14]. Apart from such deviations, the consequences of FDR are the same as of mitosis: two daughter nuclei containing the somatic number of chromosomes. Fusion of thus produced gametes with unreduced chromosome numbers produces progeny with doubled chromosome number, creating a fertile polyploid.

In standard meiosis, the basic unit of division in meiosis I is a bivalent, a structure composed of two homologues connected by a chiasma or chiasmata. Each homologue is fully replicated, so a bivalent consists of four chromatids: two sisters and two non-sisters. Dissolution of sister chromatid cohesion along the chromosome arms, but not in the centromeres, releases chiasmata, and the homologues are free to migrate to the opposite poles of the karyokinetic spindle. Separation of sister chromatids at the centromeres takes place in anaphase II. With four chromatids present, a bivalent contains four centromeres and care has to be taken to make sure the centromeres of sister chromatids (sister centromeres) co-orient to the same pole of the karyokinetic spindle. This is accomplished by different packaging of chromosomes for meiosis than for mitosis. In meiosis, sister chromatids are tightly bound by cohesins along their entire lengths, and sister centromeres are fused into single units. Hence, a bivalent presents only two centromeric units to the microtubules emanating from the poles [15,16,17]. This meiosis-specific behavior of sister centromeres, which makes orderly separation first of bivalents in anaphase I and then sister chromatids in anaphase II, is accomplished by a step-wise control of meiotic kinases [18]. However, in hexaploid wheat, univalents, which clearly are packaged in the same manner as chromosomes involved in chiasmate associations, sometimes remain on the metaphase plate and separate sister chromatids already in the first anaphase [19]. The frequency of such precocious sister chromatid separation appears to be proportional to time spent on the metaphase plate as the proportion of univalents separating sister chromatids increases with the progression of anaphase I. Most often, however, univalents in hexaploid wheat show all evidence of monopolar interaction with the karyokinetic spindle, meandering between the poles as a consequence of transient microtubule stabilization, and when off the plate, do not separate sister chromatids. On the other hand, univalents in bipolar attachment to the karyokinetic spindle are always located on the metaphase plate and often undergo misdivision (breakage across the centromere) [19].

Another striking difference in univalent behavior between standard meiosis and FDR is their orientation relative to the metaphase plate, or the axis of the karyokinetic spindle. In metaphase I of standard meiosis, univalents in a bipolar attachment to microtubules are almost always in a parallel orientation to the spindle axis (perpendicular to the metaphase plate) while those in a monopolar attachment are almost always V-shaped, probably as a consequence of friction with the cytoplasm during random meandering between the spindle poles, and the majority of them are located off the plate (on fixed preparations). Univalents left on the metaphase plate in anaphase I appear immobilized, are always oriented parallel to the plate and are straight. In FDR, univalents which congregate on the metaphase plate in MI are oriented parallel to it and are almost always straight.

In FDR, sister centromeres separate in what is the equivalent of the first meiotic division; it is an interesting question as to whether chromosomes, and specifically their centromeres, are pre-packaged for FDR differently than they are for standard meiosis, to facilitate orderly congregation on the metaphase plate, or is the packaging the same, and the conversion occurs during the division itself. Packaging similar to that for mitosis would easily explain the behavior of univalents on the FDR metaphase I plate. Mitotic-like organization, with sister centromeres on the opposite sides of replicated chromosomes, would greatly facilitate their bipolar attachment to the karyokinetic spindle, and hence, placement on the metaphase plate. However, if packaging of centromeres in FDR is the same as for normal meiosis, it is not clear how univalents with functionally single centromeres (fused sister centromeres), hence a strong propensity for monopolar interactions with the karyokinetic spindle and haphazard movements throughout the volume of a meiocyte, place themselves on the metaphase plate, attach amphitelically to the spindle, and do not appear to suffer from centric misdivision.

This study focused on the appearance of centromeres from the earliest identifiable stages of meiosis through anaphase I in wheat x rye (*Secale cereale* L.) F_1_ hybrids with FDR. The observations showed that while longitudinal differentiation along the centromeres can be observed in some chromosomes, prior to metaphase I there was no separation of sister centromeres. Such separation appeared to take place on the metaphase I plate itself. This brought about another question, so far unanswered, as to what mechanism delivers these univalents to the plate itself, and in parallel orientation to it. This parallel orientation implies that it may not be the centromeres, or not centromeres alone, that are responsible for the movement of chromosomes.

## 2. Results

### 2.1. Types of Meiotic Division

**Figure 1:**
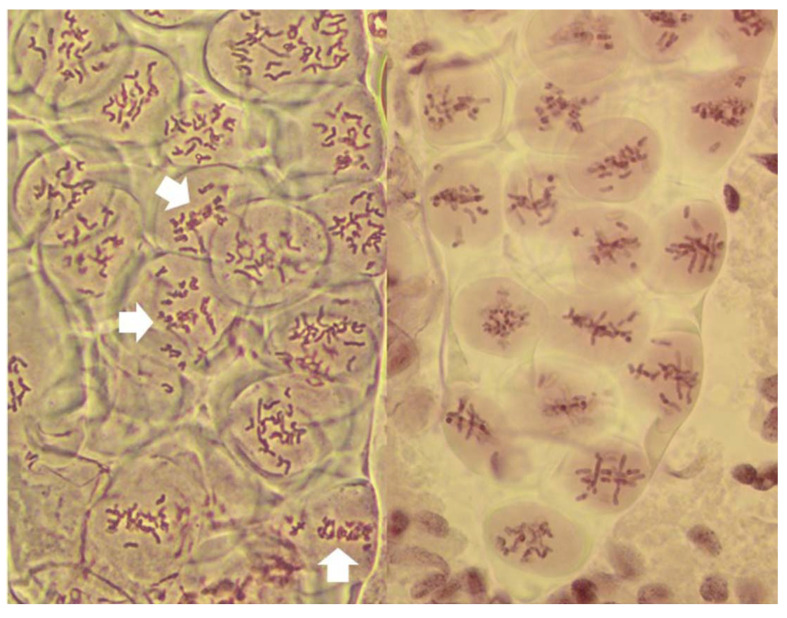
Two columns of PMCs from live stained anthers of plants undergoing FDR, at metaphase I. On the left, most PMCs undergo normal reductional meiosis, with chromosomes scattered throughout the volume of each. Three PMCs are undergoing FDR (arrows). Note that sister chromatids are clearly visible in these cells. On the right, a fragment of a column of PMCs with each cell undergoing FDR, with up to three bivalents per PMC.

**Figure 2:**
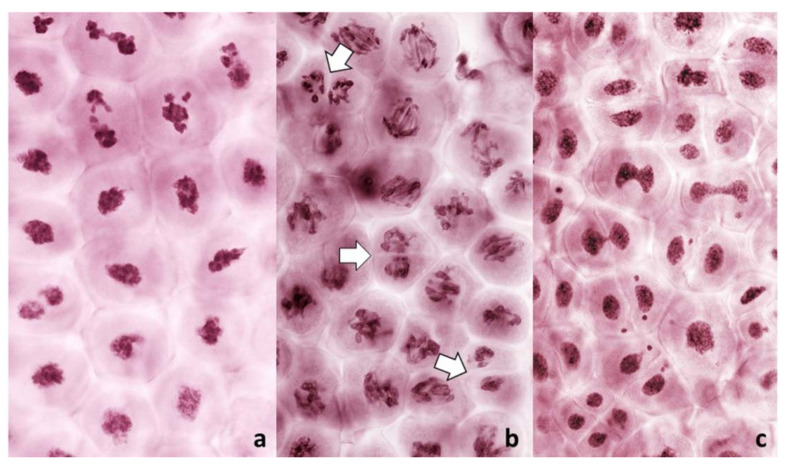
Live stained columns of PMCs undergoing an unusual division in anthers with FDR in a wheat–rye F_1_ hybrid. (**a**) early stage of division; abnormal condensation of chromatin; (**b**) mid-stage, poorly condensed chromatin, pulled apart by forces different from standard karyokinetic spindle; in some PMCs cleavage cuts through undivided nuclei (arrowed); (**c**) late stage corresponding to the tetrad stage; monads, dyads and tetrads are present, frequent thick chromatin bridges, and micronuclei.

At the MI stage, three distinct pathways of meiosis were observed: standard reductional meiosis, FDR, and the absence of normal nuclear division. The first two modes were present in varying proportions in individual anthers of all analyzed plants (Figure 1). Absence of nuclear division was observed in anthers of only three plants, and in low proportions of meiocytes (Figure 2).

**Figure 3:**
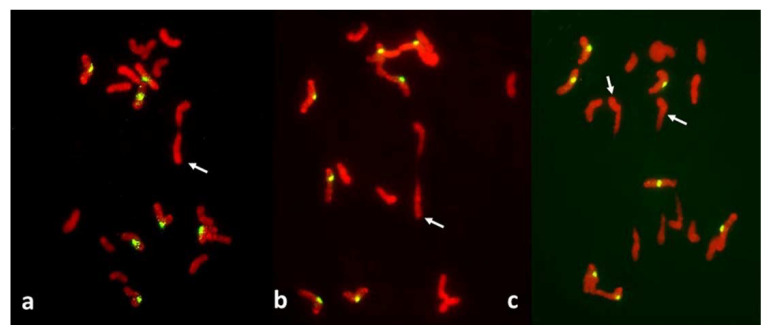
Metaphase I in PMCs with reductional meiosis in an F_1_ wheat–rye hybrid. Rye centromeres labeled green; all chromatin counterstained red. (**a**) one bivalent; nine univalents at one pole; ten univalents at the other. (**b**) one bivalent, three univalents still on the metaphase plate in a haphazard positions. (**c**) two (perhaps three) bivalents; one univalent on the metaphase plate and parallel to it, perhaps in a bipolar attachment to the spindle. All rye univalents have fused sister centromeres. Bivalents are arrowed.

**Figure 4:**
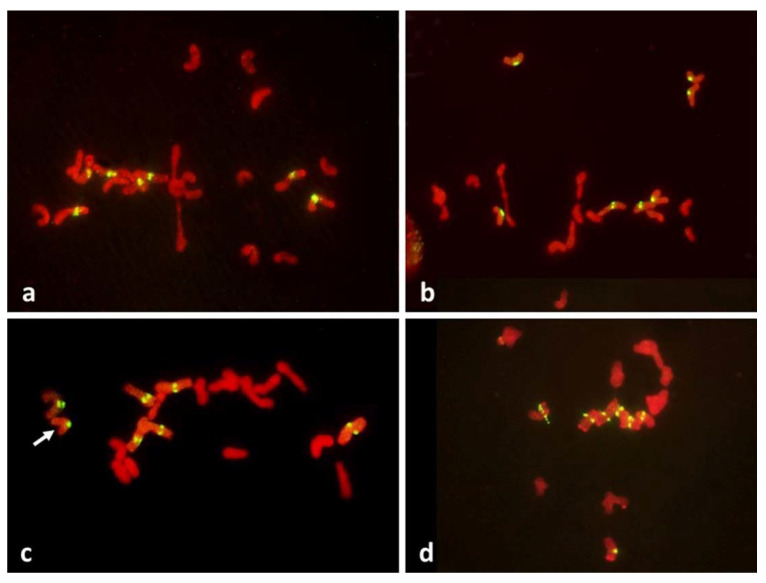
Metaphase I in PMCs with FDR in an F_1_ wheat–rye hybrid. Rye centromeres labeled green; all chromatin counterstained red. (**a**) one bivalent present; most univalents congregate on the metaphase plate; all rye univalents have fused sister centromeres; (**b**) two bivalents; four rye univalents on the metaphase plate; two with fused and two with separated sister centromeres. All three rye univalents at the pole have fused sister centromeres; (**c**) one bivalent; univalents congregating on the metaphase plate; six rye univalents with separated sister centromeres; one may still have fused sister centromeres (arrowed); it appears to be in a bipolar attachment to the karyokinetic spindle; (**d**) six rye univalents on the metaphase plate with separated sister centromeres; one precocious rye univalent at the pole, with fused sister centromeres.

**Figure 5:**
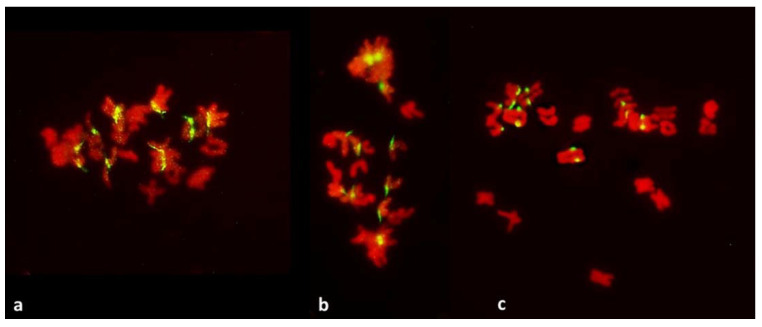
Metaphase-anaphase transition in PMCs with FDR in an F_1_ wheat–rye hybrid. Rye centromeres labeled green; all chromatin counterstained red. All rye univalents on the metaphase plate (**a**–**c**) have separated sister centromeres and are in bipolar attachment to the karyokinetic spindle. Note that univalents off the metaphase plate (five such univalents in (**c**)) have sister chromatids fused at the centromeres; none of those on the plate do.

In the standard pathway, that of regular reductional meiosis, there was some chromosome pairing (about 0.6 bivalents per PMC; only one trivalent was observed in the entire study); most chromosomes were univalent and in MI they were scattered randomly throughout the PMCs, with a tendency to concentrate at the poles (Figure 1 and Figure 3). These PMCs underwent the second division producing tetrads, usually with uneven sized nuclei and with micronuclei present. The second pathway was a typical FDR with all or most univalents concentrating on the metaphase plate (Figure 4) and separating sister chromatids while still on the metaphase plate (Figure 5). In this version of meiosis some chromosome pairing was also taking place, allowing precise stage identification, and precocious migration of some univalents to the poles was relatively frequent.

**Figure 6:**
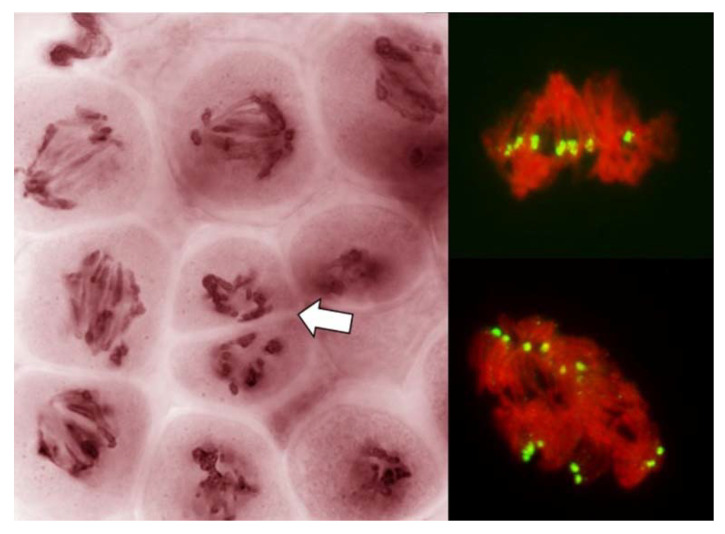
Absence of a normal nuclear division in PMCs in anthers with FDR in an F_1_ wheat–rye hybrid. A live-stained section of a column of PMCs on the left; two representative nuclei on the right after labeling with a rye-centromere specific DNA probe (green). Chromosomes are being pulled apart by unknown forces, as both figures on the right illustrate, pulling is not by the centromeres; centromeres appear to resist separation even as separation of sister centromeres is evident. Arrow points to a cell wall cutting across an undivided nucleus.

**Figure 7:**
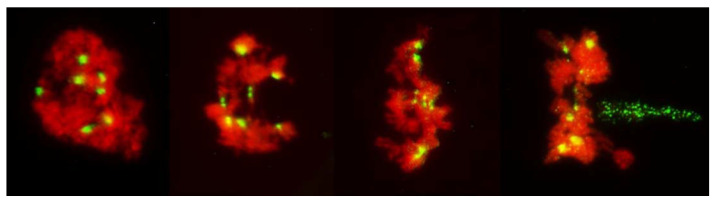
Absence of a normal nuclear division in PMCs in anthers with FDR in an F_1_ wheat x rye hybrid. Rye centromeres are labeled green; chromatin stained red. Abnormal condensation of chromatin; sister centromeres away from the tension zone are fused; those under tension are separated but appear to resist separation of chromatids. On far right, phragmoplast (green) approaches the nucleus split into two parts.

In the third pathway, precise gauging of the stage was impossible; it could only be guessed by the behavior of the near-by meiocytes undergoing one of the other two modes of division. The frequency of PMCs in this mode of division was relatively low, and they could be recognized only in later stages of the prophase, primarily by the absence of normal chromatin condensation (Figure 2). In later stages, which escape definition, uncondensed or poorly condensed chromatin appeared to be under tension and that tension did not appear to be applied to the centromeres (Figure 6 and Figure 7). Generally, these nuclei either did not undergo any organized nuclear division and formed monads, or the divisions (rather, attempts to separate chromatin) were incomplete generating large chromatin bridges (Figure 2). At times, cell cleavage cut across such undivided, or attempting to divide, nuclei (Figure 2 and Figure 7).

The presence of the three types of meiotic division was sectorial and in no case a large part of a single column of PMC in a given anther observed after live staining uniformly underwent only one type of the division. Given the nature of the preparations no firm frequencies of each of the division type could be established. In observed sections of the PMC columns, any given type of division was present from single cells to large bands of cells in a column (Figure 1 and Figure 2), and these proportions were different in different anthers from the same spikelet and spike of a plant. The fact that in each case the phenomena were sectorial served as a control in pre-MI stages, essentially guaranteeing that on any given preparation, PMCs undergoing at least two types of meiosis were present.

With the three modes of division present, the F_1_ hybrids produced microspores and pollen of three distinct sizes (not shown), most likely corresponding to haploid (or, rather, reduced number of chromosomes), diploid (that is, somatic or unreduced chromosome number) and doubled, with twice the somatic chromosome number. Given the irregularities in each type of division, some microspores and pollen grains were showing in-between sizes, but these were mostly empty.

### 2.2. Centromere Packaging in Prophase I

Individual stages of the meiotic prophase are defined by cytologically identifiable aspects of chromosome behavior. In absence of pairing, clear discrimination is not possible; here individual stages were gauged based on similarities to standard meiosis. Because of the inherent ambiguity in stage identification, for the purpose of this study the meiotic prophase was divided in three intervals, with likely overlap: leptotene-zygotene, zygotene-pachytene, and diplotene-diakinesis. Metaphase I and anaphase I were readily recognizable in normal reductional and the FDR modes of division. Gauging of stages in the absence of normal condensation of chromatin was not possible, until cytokinesis.

**Figure 8:**
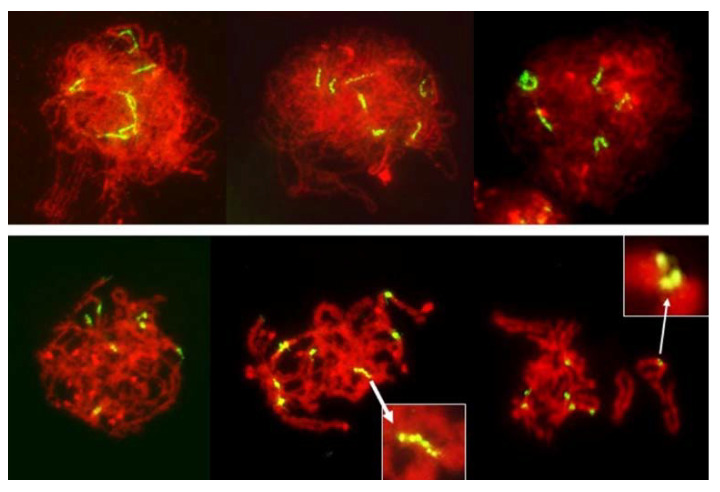
Leptotene to diplotene-like stages in an F_1_ wheat–rye hybrid. Rye centromeres labeled green; all chromatin counterstained red. Centromeres in early prophase are long and progressively contract into tight globular structures. Longitudinal differentiation of centromeres gradually disappears as chromosomes condense but occasionally it can be still be seen in pachytene/diplotene/diakinesis (insets). No separation of sister centromeres was evident in any PMC observed.

In total, 1285 PMCs in pre-MI stages were examined, of which 853 were gauged to be in the leptotene-zygotene; 241 in zygotene-pachytene, and 191 in diplotene-diakinesis. As each PMC contained seven rye chromosomes with labeled centromeres, the total number of observed centromeres was 8995. Of these, only eight produced multiple signals, all of which appeared to show longitudinal differentiation along fused centromeres. Such longitudinal differentiation was evident in the early stages of prophase (Figure 8) and it gradually disappeared as chromosome condensation progressed. In none of the pre-MI stages there were any indications of separation of PMCs into two distinct classes, with different modes of centromere packaging. Packaging and condensation of centromeres was uniform in all pre-MI PMCs scored (Figure 8).

### 2.3. Centromere Orientation at MI-AI

Among 587 PMCs examined in MI (Figure 3, Figure 4 and Figure 5), there were 344 wheat–wheat bivalents, four wheat–rye bivalents, two rye-rye bivalent, and one wheat–wheat–rye trivalent, for the average pairing of 0.6 paired chromosome arms per PMC. A single anther from plant #10 was exceptional with 233 paired arms among 111 PMCs scored, including one wheat–rye and both rye-rye bivalents, for the average of 2.1 paired arms per PMC. Several wheat–wheat bivalents were rings. Three distinct classes of MI PMCs were present: those undergoing standard reductional meiosis, those with FDR, and those with abnormal chromatin condensation and no organized nuclear division. The proportions of the FDR PMCs in individual anthers ranged from about 25% to 75%, with as much variation among anthers from a single plant as among different plants. PMCs with no organized nuclear division were observed in only three plants of the ten plants analyzed, and with low frequency. Discrimination between the FDR and reductional meiosis was based primarily on the position of univalents; univalents in the reductional meiosis tended to group at the poles; those left on the metaphase plate were usually in random orientations relative to the poles and the plate itself (Figure 3). In the FDR pathway, a majority or all univalents congregated on the metaphase plate and were oriented parallel to it (Figure 4). No separation of sister centromeres was observed in any rye univalents in the reductional meiosis. In the FDR meiosis, no univalent located away from the metaphase plate showed separation of sister centromeres; such separation was evident among a majority of univalents on the metaphase plate itself (Figure 4).

There was some relationship between chromosome pairing in MI and the mode of meiosis and hence, separation of sister centromeres in rye univalents. In one of the anthers, among 77 PMCs scored in detail, 20 had one rod bivalent each, all wheat–wheat, and two of 140 rye univalents present appeared to have separated sister centromeres. All 20 PMCs appeared to undergo standard reductional meiosis. In remaining 57 PMCs with no chromosome pairing, among 399 rye univalents present, 290 showed clear separation of sister centromeres and all PMCs appeared to undergo the FDR. However, homoeologous pairing was not a complete hindrance to FDR, in many cases a majority of univalents were arranged on the metaphase plate, ready for separation of sister chromatids, even with one or two bivalents present (Figure 4).

### 2.4. Sister Centromeres in No-Division PMCs

**Figure 9:**
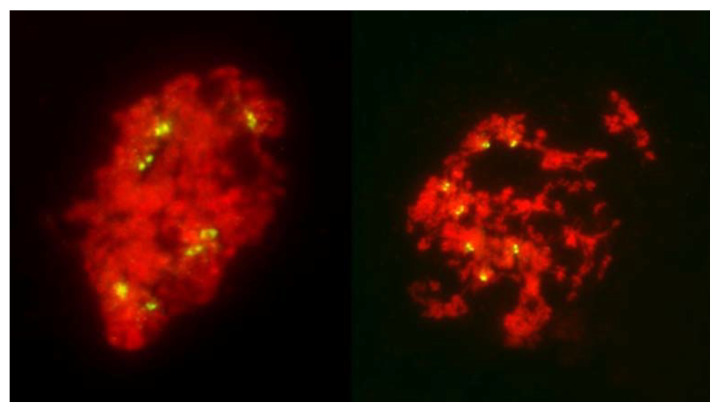
No division meiosis in an F_1_ wheat–rye hybrid; two nuclei in the stage corresponding to tetrads, which have undergone no chromatin condensation or any form of division, nuclear or cellular, and formed monads. Clear separation of sister centromeres in the absence of any pulling forces is evident.

Early on, only seven centromere signals were visible in PMCs appearing to be in the no-division pathway. At the stages where the nuclei were under clear tension, separation of sister centromeres was evident, first in the tension zone itself (Figure 6 and Figure 7) and later, in non-divided nuclei, in all seven rye chromosomes (Figure 9). However, in all cases sister centromeres were side-by side, as clearly there was no separation of sister chromatids and the tension to chromatin was not applied through the centromeres.

## 3. Discussion

There was nothing unusual about three different modes of meiotic division, in most cases side-by-side in individual anthers, observed in the material analyzed here; it has been observed in similar hybrids of wheat before [1,4,6,20]. Here, it served as built-in control for the FDR; the frequencies of normal reductional meiosis and of FDR were high in all tested plants guaranteeing that samples observed in the pre-MI stages contained PMCs destined for one of the three pathways. The no-division meiosis was the least frequent, and perhaps for that reason was observed in only three of the ten analyzed plants.

It has been proposed that FDR was delayed relative to normal meiosis [21]. In standard reductional meiosis, univalents lingering on the metaphase plate frequently separate sister chromatids in AI, and this frequency increases with time spent on the plate [19].). In this sense, more time spent on the metaphase plate in FDR would explain sister chromatid separation in AI. This notion is well supported by published images such as Figure 1e in [20] where a PMC undergoing FDR is surrounded by PMCs in MII-AII (that is, PMCs undergoing standard reductional meiosis). However, in this study, quite the opposite was observed, and with considerable frequency: in a column of PMCs in a typical MI stage both types of division, reductional and FDR, are present side-by-side (Figure 1). As a matter of fact, because the FDR PMCs clearly show separation of sister chromatids, effectively they are already in the AI-like stage while PMCs with reductional division are still in MI, because bivalents are present. Therefore, the FDR PMCs appear to be ahead of the reductional division PMCs. On the other hand, the no-division PMCs clearly are delayed relative to reductional meiosis (and FDR) but the fault here appears to be in chromatin condensation; it is only partial, no metaphase-like chromosomes are ever formed, and there is no nuclear division, even if cytokinesis may proceed.

It is evident from the observations, on large numbers of PMCs and univalents, that packaging of centromeres was the same in all observed prophase I PMCs, regardless of their later mode of division: the FDR with essentially mitotic-like separation of sister chromatids, a standard reductional meiosis or no division at all. There is a clear difference between mitotic and meiotic packaging of chromosomes, including their centromeres, which makes separation of chromosomes from bivalents, and later of sister chromatids, possible [22]. In yeast, the mono-orientation of sister kinetochores in meiosis involves a meiosis-specific cohesin subunit [23] which makes the two-step dissolution of cohesion possible [24]. This meiosis-specific cohesion subunit is protected in the first meiotic division, guaranteeing co-migration of sister chromatids to daughter nuclei, and dissolved only in the second division. Here, in all pre-MI PMCs observed, all sister centromeres were fused into single units from the earliest recognizable stages of the meiotic prophase until metaphase I (Figure 8) offering no hint of differences in packaging for different modes of meiosis. Reorganization of sister centromeres in univalents occurred only during MI (bivalents were still present) on the MI plate itself, and only in the PMCs undergoing FDR. This reorganization appeared to take place only in univalents located on the plate itself, or in its immediate vicinity, and never occurred in paired chromosomes. Univalents which precociously moved away from the plate were immune to change, and remained with their sister centromeres fused (Figure 4 and Figure 5). Very similar observations were described in [25], in a similar material and using a similar approach: univalents with labeled centromeres approach the metaphase plate amphitelically oriented toward the spindle poles, and perpendicular to the spindle axis, and eventually separate sister chromatids. As in this study, figures in [25] show no indication of sister centromere separation which would facilitate such an orderly migration.

**Figure 10:**
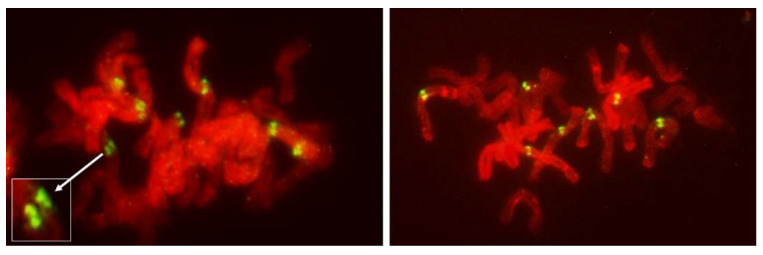
Untreated mitotic metaphase in somatic tissue of an FDR anther in an F_1_ wheat–rye hybrid. Rye centromeres labeled green; all chromatin counterstained red. Evident separation of sister centromeres in each of the seven rye chromosomes. In some cases (inset) globular nature of individual centromeres is evident.

In a standard nuclear division, be it mitosis or meiosis, placement of a chromosome on the metaphase plate is a consequence of a bipolar/amphitelic attachment of the centromeres, with their kinetochores, to the karyokinetic spindle. In mitosis, a replicated chromosome presents two centromeres to the karyokinetic spindle; these sister centromeres are on the opposite sides of a replicated chromosome, and in a constriction which further restricts the angle from which each one can be targeted by microtubule fibers. This promotes proper bipolar interaction with microtubule fibers which stabilizes the chromosome on the metaphase plate, with each centromere facing one of the spindle poles (Figure 10). A bivalent in meiosis also presents two centromere units (each with fused sister centromeres) to the karyokinetic spindle and when properly attached, becomes stabilized on the metaphase plate, oriented perpendicular to it. For univalents, a bipolar attachment of fused sister centromeres also places them on the metaphase plate, and also orients them perpendicular to the plate. This often leads to centric misdivision (see Figure 2 in [19]). Only when such univalents resist misdivision and linger on the plate well into the anaphase, separation of sister centromeres takes place: they reorient parallel to the (former) metaphase plate, and eventually separate sister chromatids. Clearly, in wheat both meiotic steps of sister chromatid separation can take place in the first division [19].

Occasionally, fixation may catch on the metaphase plate a meandering univalent with a monopolar attachment to the karyokinetic spindle, but these are fairly infrequent and easily recognizable events. Univalents in a monopolar attachment are almost always V-shaped (for two-armed chromosomes) and apart from extended centromeres, generally show no signs of tension. In this context, striking was the difference between the FDR and reductional meiosis in the disposition of univalents in a PMC. In MI of reductional meiosis (bivalents are present, Figure 3), a majority of univalents congregated close to the poles of the karyokinetic spindle, none showed any sign of sister centromere separation and all had fused sister chromatids. Most were either V-shaped or lay parallel to the axis of the spindle. In the FDR meiocytes at MI (as indicated by the presence of bivalents), most univalents congregated on the metaphase plate or close to it, still with sister centromeres fused, but generally straight and perpendicular to the spindle axis (parallel to the metaphase plate). While on the plate itself, sister centromeres separated, at times when sister chromatids still appeared to be attached (Figure 5). At the onset of anaphase, univalents away from the plate clearly showed separation of sister chromatids along the length of the arms while sister centromeres remained fused (Figure 5a,c) while those on the plate were showing essentially the opposite.

Differences in univalent orientation and their centromere status imply that perhaps it is not the karyokinetic spindle that places them on the metaphase plate, or that it is not an interaction between the spindle and the centromeres. Cai et al. [5] have shown that at this stage in the FDR, fully developed karyokinetic spindle is present. Here, even though the microtubules of the karyokinetic spindle were not visualized, it was evident that normally functional karyokinetic spindle also had to be present: bivalents were property positioned on the metaphase plate and were stretched; univalents away from the plate showed all signs of monopolar attachment to the spindle and of the movement dictated by such attachment. However, in the FDR PMCs, univalent behavior and orientation were different from that in standard reductional meiosis or in mitosis. It is not clear what mechanism/system may be responsible for the delivery of univalents to the metaphase plate in the FDR meiocytes in a fashion clearly different from the normal monopolar or bipolar centromere-spindle interaction. That some other mechanism of chromosome movement/transport may be at play is suggested by the appearance and behavior of chromatin in no-division PMCs: clearly, something attempts to separate that chromatin (mostly uncondensed or poorly condensed) and it is not by the attachment to the centromeres but to other parts of chromosomes (Figure 6 and Figure 7) as with some frequency the centromeres/centromere regions still provide cohesion of sister chromatids and appear to resist the separation (Figure 7). Perhaps these are the abnormal karyokinetic spindles as described by [20,26,27]. However, here this mechanism appears to operate simultaneously with a normally functional spindle apparatus, as indicated by perfectly normal positioning of bivalents, and univalents off the metaphase plate (V-shaped, clearly caught in transit through the cytoplasm). In large cells such as oocytes in amphibians, where the centromeres may be beyond the reach/range of microtubules there is an alternative system which delivers chromosomes to the spindle [28]. Here, however, the situation is different: all univalents clearly are within the range of the spindle but most of them do not appear to interact with it in a standard way in FDR PMCs.

It could be argued that in the absence of pulling forces, separation of sister centromeres would be difficult to detect or be outright undetectable in the pre-MI stages. However, MI itself is informative, and so are several other configurations. In the MI stage of FDR, univalents congregate on the metaphase plate with their sister centromeres fused; these centromeres separate on the metaphase plate itself at which point their bipolar attachment, and the presence of pulling forces, is evident. At the same time and in the same PMCs, univalents beyond the plate still retain their centromere-fused configuration even when cohesion of sister chromatids away from the centromere is already released. This is in clear contrast to the mitotic configuration: in somatic cells of anthers with FDR undergoing standard mitosis (karyokinetic spindle is present) where all labeled centromeres on the metaphase plate are separated (Figure 10). They are also separated in nuclei undergoing no division meiosis at the stage corresponding to tetrads, where no pulling forces appear to exist (Figure 9).

## 4. Materials and Methods

All samples for this study were collected during the earlier study of the first meiotic restitution in an F_1_ wheat–rye hybrid [14]. The plant material consisted of ten F_1_ hybrids of a tetraploid wheat line Do1 with a population rye *Secale cereale* L. designated as MAD510. The Do1 line was selected by Dr. B. Lapinski, then at the Institute of Plant Genetics, Poznan, Poland, from among the hybrids of *Triticum turgidum var. dicoccoides ssp. spontaneonigrum x T. turgidum var. persicum*. The selection criterion was its ability to produce fully fertile F_1_ hybrids with rye, a characteristic later identified as a consequence of the restitution of the first meiotic division (FDR). MAD510 was created by an intercross of winter rye cultivars Motto, Amilo and Dankowskie Zlote, all originating from the Danko Plant Breeding, Choryn, Poland; the population is homozygous for an introgression of a segment of wheat chromosome 1D with the locus *Glu-D1* [29].

The F_1_ hybrids were grown in a greenhouse at the University of California, Riverside CA campus. Tillers with spikes judged to be at meiosis were cut, spikes dissected and single anthers from individual spikelets were squashed in a drop of acetocarmine. If the desired stage of meiosis was present the remaining two anthers were fixed in a mixture of absolute ethyl alcohol and glacial acetic acid, in proportion 3:1, respectively, and stored at −20 °C for analyses.

Some observations were made on live-stained anthers during collection of the material. These observations were limited to a gross assessment of the proportions of pollen mother cells (PMCs) in various modes of meiosis. Observations of the centromeres were done on squash preparations from fixed anthers, probed in situ with DNA of the rye centromere sequence [30] labeled with DIG-oxygenin using the DIG-Nick Translation Kit (Roche) according to manufacturer’s instructions, and hybridized to chromosome preparations according to the protocol in [31]. Hybridization signals were detected with anti-DIG-FITC; herring DNA was used as a block, usually in about 50x excess relative to the probe. Counterstaining was done with 0.3% propidium iodide in the Vectashield antifade solution. Observations were made under a Zeiss Axioscope 20 equipped with epi-fluorescence, recorded with a SPOT RT Color digital camera (Diagnostic Instruments Inc., Sterling Heights, MI, USA ), and processed using the SPOT Advanced and Adobe Photoshop CS software. All images presented here were manipulated as needed to enhance contrast, remove debris if present, properly orient them on the N-S axis and tone down the background noise.5.

## 5. Conclusions

This study indicates that in FDR centromeres are not specifically pre-packaged for separation of sister chromatids in AI. Separation of sister centromeres, which makes FDR possible, takes place on the metaphase plate itself, and within a very limited distance from that plate. Univalents away from the plate retain sister centromere fusion and contribute both sister chromatids to one of the nuclei of the dyad. Interactions of univalents with the karyokinetic spindle appear to be an unlikely mechanism responsible for an orderly congregation of univalents on the metaphase plate; this would require bipolar attachment to the spindle and for the observed orientation of univalents (parallel to the plate) separation of sister centromeres appears necessary. In standard (reductional) meiosis, separation of sister centromeres of univalents in MI is rare; it increases only with time spent on the plate in AI. Rare univalents with separated sister centromeres in MI of normal meiosis are oriented parallel to the plate as are all those left on the metaphase plate in AI. On the other hand, univalents with fused sister centromeres in MI of normal meiosis either are located off the plate, caught by fixation in the process of migration toward one of the poles because of the monopolar attachment; those with bipolar attachment are on the metaphase plate and oriented perpendicular to the plate. These are subject of misdivision. In the system observed here, separation of sister centromeres is not required for the on-the-plate positioning of univalents.

## Data Availability

All pertinent data are included in this article. Additional information, if needed, can be obtained from the author.

## References

[1] Xu S.J., Joppa L.R. (2000). First division restitution in hybrids of Langdon durum disomic substitution lines with rye and Aegilops squarrosa. Plant Breed..

[2] Matsuoka Y., Nasuda S. (2004). Durum wheat as a candidate for the unknown female progenitor of bread wheat: An empirical study with a highly fertile F1 hybrid with Aegilops tauschii Coss. Theor. Appl. Genet..

[3] Jauhar P.P. (2007). Meiotic restitution in wheat polyhaploids (amphihaploids): A potent evolutionary force. J. Hered..

[4] Zhang L.Q., Yen Y., Zheng Y.L., Liu D.C. (2007). Meiotic restriction in emmer wheat is controlled by one or more nuclear genes that continue to function in derived line. Sex. Plant Reprod..

[5] Cai X., Xu S.S., Zhu X.X. (2010). Mechanism of haploidy-dependent unreductional meiotic cell division in polyploid wheat. Chromosoma.

[6] Silkova O.G., Shchapova A.I., Shumny V.K. (2011). Meiotic restitution in amphiploids in the tribe Triticeae. Rus. J. Genet..

[7] Bretagnole F., Thomson J.D. (1995). Gametes with the somatic chromosome number: Mechanisms of their formation and role in the evolution of autopolyploid species. New Phytol..

[8] Ramana M.S., Jacobsen E. (2003). Relevance of sexual polyploidization for crop improvement—A review. Euphytica.

[9] Lim K.B., Ramana M.S., DeJong J.H., Jacobsen E., Van Tuyt J.M. (2001). Indeterminate meiotic restitutions (IMR): A novel type of meiotic nuclear restitution mechanism detected in interspecific lily hybrids by GISH. Theor. Appl. Genet..

[10] Wang C., Zhang L., Dai S.F., Zheng Y.I., Zhang H.G., Liu D.C. (2010). Formation of unreduced gametes is impeded by homologous chromosome pairing in tetraploid Triticum turgidum × Aegilops tauschii hybrids. Euphytica.

[11] Kynast R.G., Davis D.W., Philips R.I., Rines H.W. (2012). Gamete formation via meiotic nuclear restitution generates fertile amphiploid F 1 (oat x maize) plants. Sex. Plant Reprod..

[12] Chester M., Gallagher J.P., Symonds V.V., Sa Silvac A.V.C., Mavrodiev E.V., Leitch A.R., Soltis P.S., Soltis D.E. (2021). Extensive chromosomal variation in a recently formed natural allopolyploid species, Tragopogon miscellus (Asteraceae). Proc. Natl. Acad. Sci. USA.

[13] Zhang H., Bian Y., Gou X., Shu B., Xu C., Qi B., Li N., Rustgi S., Zhou H., Han F. (2013). Persistent whole-chromosome aneuploidy is generally associated with nascent allohexaploid wheat. Proc. Natl. Acad. Sci. USA.

[14] Oleszczuk S., Lukaszewski A.J. (2014). The origin of unusual chromosome constitutions among newly formed allopolyploids. Am. J. Bot..

[15] Dawe R.K. (1998). Meiotic chromosome organization and segregation in plants. Ann. Rev. Plant Phys. Plant Mol. Biol..

[16] Li X., Dawe R.K. (2009). Fused sister kinetochores initiate the reductional division in meiosis I. Nat. Cell Biol..

[17] Miller M.P., Amon A., Unal E. (2013). Meiosis I: When chromosomes undergo extreme makeover. Curr. Opin. Cell Biol..

[18] Galander S., Barton R.E., Borek W.E., Spanos C., Kelly D.A., Robertson D., Rappsilber J., Marston A.L. (2019). Reductional meiosis I chromosome segregation is established by coordination of key meiotic kinases. Dev. Cell.

[19] Lukaszewski A.J. (2010). Behavior of centromeres in univalents and centric misdivision in wheat. Cytogenet. Genome Res..

[20] Silkova O.G., Loginova D.B. (2016). Sister chromatid separation and monopolar spindle organization in the first meiosis as two mechanisms of unreduced gametes formation in wheat-rye hybrids. Plant Reprod..

[21] Hao M., Luo J., Zeng D., Zhang L., Ning S., Yuan Z., Yan Z., Zhang H., Zheng Y., Feuillet C. (2014). QTug.sau-3B is a major quantitative trait locus for wheat hexaploidization. Genes Genomes Genet..

[22] Hauf S., Watanabe Y. (2004). Kinetochore orientation in mitosis and meiosis. Cell.

[23] Watanabe Y., Nurse P. (1999). Cohesin Rec8 is required for reductional chromosome segregation at meiosis. Nature.

[24] Brar G.A., Amon A. (2008). Emerging roles for centromeres in meiosis I chromosome segregation. Nat. Genet..

[25] Zeng D.Y., Hao M., Luo J.T., Zhang L.Q., Yuan Z.W., Ning S.Z., Zheng Y.L., Liu D.C. (2014). Amphitelic orientation of centromeres at metaphase I is an important feature for univalent-dependent meiotic nonreduction. J. Genet..

[26] Shamina N., Dorogova N., Goncharov N., Orlova A., Trunova S. (1999). Abnormalities of spindle and cytokine behavior leading to the formation of meiotic restitution nuclei in intergeneric cereal hybrids. Cell Biol. Int..

[27] Shamina N.V., Silkova O.G., Seriukova E.G. (2003). Monopolar spindles in meiosis of intergeneric cereal hybrids. Cell Biol. Int..

[28] Caudron M., Bunt G., Bastiaens P., Karsenti E. (2005). Spatial coordination of spindle assembly by chromosome-mediated signaling gradients. Science.

[29] Lukaszewski A.J., Brzezinski W., Klockiewicz-Kaminska E. (2000). Transfer of the Glu-D1 locus encoding high molecular weight glutenin subunits 5+10 from breadwheat to diploid rye. Euphytica.

[30] Francki M. (2001). Identification of Bilby, a diverged centromeric Ty1-copia retrotransposon family from cereal rye (*Secale cereale* L.). Genome.

[31] Massoudi-Nejad A., Nasuda S., McIntosh R.A., Endo T.R. (2002). Transfer of rye chromosome segments to wheat by gametocidal system. Chromosome Res..

